# Baseline Characteristics and Prescription Patterns of Standard Drugs in Patients with Angiographically Determined Coronary Artery Disease and Renal Failure (CAD-REF Registry)

**DOI:** 10.1371/journal.pone.0148057

**Published:** 2016-02-09

**Authors:** Holger Reinecke, Günter Breithardt, Christiane Engelbertz, Roland E. Schmieder, Manfred Fobker, Hans O. Pinnschmidt, Boris Schmitz, Philipp Bruland, Karl Wegscheider, Hermann Pavenstädt, Eva Brand

**Affiliations:** 1 Division of Vascular Medicine, Department of Cardiovascular Medicine, University Hospital Muenster, Muenster, Germany; 2 Department of Nephrology, Hypertension, and Rheumatology, University Hospital Muenster, Muenster, Germany; 3 Department of Nephrology and Hypertension, University of Erlangen-Nuernberg, Erlangen, Germany; 4 Institute of Clinical Chemistry and Laboratory Medicine, University Hospital Muenster, Muenster, Germany; 5 Department of Medical Biometry and Epidemiology, University Medical Centre Hamburg-Eppendorf, Hamburg, Germany; 6 Institute of Sports Medicine, Molecular Genetics of Cardiovascular Disease, University Hospital Muenster, Muenster, Germany; 7 Institute of Medical Informatics, University of Muenster, Muenster, Germany; Shenzhen Institutes of Advanced Technology, CHINA

## Abstract

**Background:**

Chronic kidney disease (CKD) is strongly associated with coronary artery disease (CAD). We established a prospective observational nationwide multicenter registry to evaluate current treatment and outcomes in patients with both CKD and angiographically documented CAD.

**Methods:**

In 32 cardiological centers 3,352 CAD patients with ≥50% stenosis in at least one coronary artery were enrolled and classified according to their estimated glomerular filtration rate and proteinuria into one of five stages of CKD or as a control group.

**Results:**

2,723 (81.2%) consecutively enrolled patients suffered from CKD. Compared to controls, CKD patients had a higher prevalence of diabetes, hypertension, peripheral artery diseases, heart failure, and valvular heart disease (each p<0.001). Myocardial infarctions (p = 0.02), coronary bypass grafting, valve replacements and pacemaker implantations had been recorded more frequently (each p<0.001). With advanced CKD, the number of diseased coronary vessels and the proportion of patients with reduced left ventricular ejection fraction (LVEF) increased significantly (both p<0.001). Percutaneous coronary interventions were performed less frequently (p<0.001) while coronary bypass grafting was recommended more often (p = 0.04) with advanced CKD. With regard to standard drugs in CAD treatment, prescriptions were higher in our registry than in previous reports, but beta-blockers (p = 0.008), and angiotensin-converting-enzyme inhibitors and/or angiotensin-receptor blockers (p<0.001) were given less often in higher CKD stages. In contrast, in the subgroup of patients with moderately to severely reduced LVEF the prescription rates did not differ between CKD stages. In-hospital mortality increased stepwise with each CKD stage (p = 0.02).

**Conclusions:**

In line with other studies comprising CKD cohorts, patients’ morbidity and in-hospital mortality increased with the degree of renal impairment. Although cardiologists’ drug prescription rates in CAD-REF were higher than in previous studies, they were still lower especially in advanced CKD stages compared to cohorts treated by nephrologists.

## Introduction

Chronic kidney disease (CKD) belongs to the fastest growing and globally occurring health burdens, affecting patients at any age and across ethnic groups [1,2]. CKD determined by decreased glomerular filtration rate (GFR) or isolated proteinuria is strongly associated with severe cardiovascular diseases [3–5] such as coronary artery disease (CAD), myocardial infarction (MI), congestive heart failure, stroke, and atrial fibrillation [4,6–10]. Apart from multiple unfavorable pathophysiological causes leading to the increased morbidity and mortality in CKD in general [11,12], non-adherence in terms of standard guideline therapies, such as drugs prescriptions, and interventional measures is one additional cause for the deleterious outcome of CKD patients [13–15]. Therefore, current guidelines highlight the importance of consequent treatment especially by standard drugs [16].

Despite a vast knowledge of development and progression of CKD and CAD [11,12] individual risk stratification and new successful treatment strategies in CKD patients are still missing [17]. Therefore, a few recent studies have been established which aimed to identify new risk factors and biomarkers as well as differences in the current treatment of CKD patients contributing to their deleterious prognosis [17–21]. Here we report on the baseline characteristics and in-hospital outcomes of the *Coronary Artery Disease and Renal Failure* (CAD-REF) Registry [21] including 3,352 German patients with angiographically documented CAD and different stages of CKD including CAD patients with normal renal function as control subjects. With regard to the elsewhere reported therapeutic nihilism or so-called inertia [13–15] we also focused on drug prescriptions at enrollment and discharge in different CKD stages.

## Materials and Methods

The German CAD-REF-registry is a nationwide multicenter prospective observational registry of patients with at least one documented coronary artery stenosis ≥50% in a major coronary vessel and different degrees of CKD. Between January 2008 and May 2011, 3,352 patients were enrolled at 32 recruiting cardiological centers located all over Germany. Recruiting centers were hospitals with a department of cardiology, or internal medicine with cardiological focus, as well as cardiologists in private practices performing coronary angiographies. Patients were followed up for at least 24 month using standardized validated questionnaires. The detailed trial design has been reported elsewhere [21].

An approval was obtained from the Ethic Committee of the Landesärztekammer Westfalen-Lippe and the Medical Faculty of the Westfälische Wilhelms-University Muenster (date August 16, 2007; No 2007-315-f-S). The study was conducted in accordance with the Declaration of Helsinki. In December 2007 the contract research organization was instructed to register the protocol of the CAD-REF registry at ClinicalTrials.gov. Accidentally the registration was delayed until May 2008. The identifier number is NCT00679419 (http://clinicaltrials.gov/). The authors confirm that all ongoing and related trials for this drug/intervention are registered.

### Inclusion criteria, exclusion criteria and recruitment

In brief, patients were included if they were ≥18 years, of Western European descent (Western European parents and grandparents) and gave written informed consent. All had undergone a current diagnostic coronary angiography documenting at least one stenosis ≥50% in at least one major coronary vessel (main stem, ramus circumflexus [LCX], left anterior descending coronary artery [LAD], right coronary artery [RCA]).

Patients were included only once in the registry. Patients with organ transplantations other than kidney transplantation, with immunosuppressive therapy apart from immunosuppressive therapy after kidney transplantation, with polycystic renal disease, with known malignant tumors as well as pregnant or breastfeeding patients were excluded from the registry.

### Renal function

Serum creatinine (Scr) values were based on the method by Jaffé. To estimate the GFR, the CKD-EPI formula was used [22,23]:

female patients:

$$\text{Scr}\leq 0.7\;\text{mg}/\text{dl eGFR}\;(\text{ml}/\text{min}/1.73\;{\text{m}}^{2})=144\times {\left(\text{Scr}/0.7\right)}^{-0.329}\times {\left(0.993\right)}^{\text{age}/\text{years}}$$

$$\text{Scr}>0.7\;\text{mg}/\text{dl eGFR}\;(\text{ml}/\text{min}/1.73\;{\text{m}}^{2})=144\times {\left(\text{Scr}/0.7\right)}^{-1.209}\times {\left(0.993\right)}^{\text{age}/\text{years}}$$

male patients:

$$\text{Scr}\leq 0.9\;\text{mg}/\text{dl eGFR}\;(\text{ml}/\text{min}/1.73\;{\text{m}}^{2})=141\times {\left(\text{Scr}/0.9\right)}^{-0.411}\times {\left(0.993\right)}^{\text{age}/\text{years}}$$

$$\text{Scr}>0.9\;\text{mg}/\text{dl eGFR}\;(\text{ml}/\text{min}/1.73\;{\text{m}}^{2})=141\times {\left(\text{Scr}/0.9\right)}^{-1.209}\times {\left(0.993\right)}^{\text{age}/\text{years}}$$

Based on the results of the eGFR and the dipstick test on proteinuria, patients were classified into 5 CKD categories in accordance to the classification of the National Kidney Foundation [16] as follows: category 1: eGFR ≥90 ml/min/1.73 m² and proteinuria; category 2: eGFR 60–89 ml/min/1.73 m²; category 3: eGFR 30–59 ml/min/1.73 m²; category 4: eGFR 15–29 ml/min/1.73 m²; category 5: eGFR <15 ml/min/1.73 m². Patients on dialysis were also assigned to category 5 regardless of their eGFR. Dipstick test on proteinuria was missing in 10 patients with no residual urine.

Patients with eGFR ≥90 ml/min/1.73 m² and a negative dipstick test were classified as control patients.

### Study data collection

Data collection was performed by the IKKF Institute, Munich, Germany. Primary route of data entry was via online electronic case forms. All information was kept confidential and transmitted data was pseudonymized.

At baseline visit, demographic characteristics, anthropometric data, medical history, and ECG parameters were recorded. Initial data collection comprised a precisely characterized coronary angiography with the degree and localization of stenosis according to the Cardiology Audit and Registration Data Standards (CARDS) [24], medication, and standard serum and urine laboratory parameters. In the case of a coronary intervention, additional data concerning cardiac status and events in accordance to CARDS, indication for percutaneous coronary intervention (PCI), stent implantation, degree of stenosis, medication, contrast dye application, renal status and complications after intervention were also recorded.

### Data and statistical analysis

Standard descriptive statistical methods were used to describe baseline characteristics. Means with SD were given for continuous variables, and frequency distribution with percentages was used for categorical variables. Right-skewed variables, such as laboratory variables, were logarithmic (ln) transformed. Their means and 95% CIs intervals are presented in original scale after back-transformation. The Jonckheere-Terpstra-test (J-T-test) was applied to test for trends of ordinal and continuous variables across ordered categories. The Kruskal-Wallis test was applied for the comparison of nominal categories, with subsequent Mann-Whitney-U-tests comparing each category to all others. Non-directional associations were studied by Spearman correlation coefficients. To test the effects of CKD stage, visit and their interaction on medicalization, a logistic regression model was fit with intercept and the fixed effects CKD stage, visit and their interaction. CKD stage was treated as a categorical variable. A two-sided p≤0.05 was considered statistically significant.

## Results

Between January 2008 and May 2011, 3,352 patients who met the inclusion criteria were consecutively included into the registry without any preselection. Table 1 and S1 Fig show the patients’ characteristics and distribution for the whole study population, and in detail for the 6 subgroups.

**Table 1:** Patient characteristics at the time of enrollment.

	Overall population	Controls (eGFR ≥90 ml/min/1.73 m²), no proteinuria	CKD stage 1 (eGFR ≥90 ml/min/1.73 m²), proteinuria	CKD stage 2 (eGFR 60–89 ml/min/1.73 m²)	CKD stage 3 (eGFR 30–59 ml/min/1.73 m²)	CKD stage 4 (eGFR 15–29 ml/min/1.73 m²)	CKD stage 5 (eGFR <15 ml/min/1.73 m²) or dialysis	p
Patients, n (% of all)	3,352 (100.0)	629 (18.8)	127 (3.8)	1,599 (47.7)	854 (25.5)	107 (3.2)	36 (1.1)	
Women, n (%)	773 (23.1)	96 (15.3)	27 (21.3)	348 (21.8)	246 (28.8)	43 (40.2)	13 (36.1)	**<0.001**
Age, mean ± SD, years	67.1 ± 10.4	57.1 ± 8.6	58.5 ± 9.7	67.8 ± 9.1	73.2 ± 7.7	75.0 ± 8.5	72.3 ± 7.9	**<0.001**
Height, mean ± SD, cm	172.5 ± 8.6	174.9 ± 8.3	173.8 ± 8.0	172.9 ± 8.5	170.4 ± 8.4	168.8 ± 9.0	169.4 ± 9.1	**<0.001**
Weight, mean ± SD, kg	84.2 ± 15.1	86.0 ± 15.6	86.1 ± 15.8	84.3 ± 14.9	83.0 ± 14.8	82.2 ± 16.8	80.3 ± 11.5	**<0.001**
Proteinuria, n (%)	637 (19.1)	0 (0.0)	127 (100.0)	249 (15.6)	205 (24.0)	38 (35.5)	18 (69.2)	**<0.001**
Dyslipidemia, n (%)	2,178 (67.8)	420 (70.0)	77 (65.3)	1,048 (68.4)	546 (66.3)	66 (64.1)	21 (58.3)	0.08
Diabetes, n (%) ^a)^	1,076 (32.1)	135 (21.5)	35 (27.6)	480 (30.0)	350 (41.0)	57 (53.8)	19 (52.8)	**<0.001**
Insulin-dependent, n (%) ^b)^	427 (12.7)	47 (7.5)	15 (11.8)	155 (9.7)	156 (18.3)	37 (34.9)	17 (47.2)	**<0.001**
Tobacco use: ever smokers, n (%)	1,769 (54.3)	448 (72.5)	84 (68.3)	796 (51.5)	383 (46.1)	46 (44.2)	12 (34.3)	**<0.001**
Alcohol consumption, n (%)	1,611 (59.0)	355 (65.7)	82 (72.6)	787 (60.6)	349 (51.4)	29 (39.2)	9 (32.1)	**<0.001**
PAOD, n (%)	350 (10.5)	31 (4.9)	12 (9.4)	143 (9.0)	121 (14.2)	29 (27.4)	14 (40.0)	**<0.001**
Family history CHD, n (%)	1,176 (42.5)	263 (50.6)	61 (56.0)	566 (43.7)	252 (34.4)	28 (32.2)	6 (26.1)	**<0.001**
Arterial hypertension, n (%)	2,794 (83.4)	450 (71.5)	99 (78.0)	1,329 (83.2)	781 (91.5)	102 (95.3)	33 (91.7)	**<0.001**
Pulse pressure, mean ± SD, mmHg	57.6 ± 16.4	54.0 ± 14.5	56.1 ± 15.4	58.9 ± 16.5	58.1 ±16.9	58.7 ± 18.0	57.7 ± 19.6	**<0.001**
Left ventricular hypertrophy*, n (%)	95 (2.9)	19 (3.0)	13 (10.3)	34 (2.1)	21 (2.5)	6 (5.8)	2 (5.7)	0.46
Valvular heart disease, n (%)	454 (13.5)	32 (5.1)	13 (10.2)	201 (12.6)	172 (20.1)	27 (25.2)	9 (25.0)	**<0.001**
Aortic valve stenosis, n (%)	185 (41.1)	8 (25.0)	5 (38.5)	72 (36.2)	82 (48.2)	16 (59.3)	2 (22.2)	**0.003**
Previous stroke, n (%)	188 (5.6)	20 (3.2)	5 (3.9)	86 (5.4)	66 (7.7)	6 (5.7)	5 (13.9)	**<0.001**
Previous MI, n (%)	1,086 (32.5)	214 (34.0)	26 (20.5)	484 (30.4)	299 (35.0)	49 (45.8)	14 (38.9)	**0.02**
Previous CABG, n (%)	682 (20.3)	78 (12.4)	12 (9.4)	299 (18.7)	248 (29.0)	34 (31.8)	11 (30.6)	**<0.001**
Previous PCI, n (%)	1,494 (44.6)	271 (43.2)	33 (26.0)	717 (44.8)	402 (47.1)	57 (53.3)	14 (40.0)	**0.006**
Previous valve replacement, n (%)	48 (1.4)	4 (0.6)	1 (0.8)	18 (1.1)	16 (1.9)	5 (4.7)	4 (11.1)	**<0.001**
Pacemaker, n (%)	232 (6.9)	13 (2.1)	5 (3.9)	81 (5.1)	112 (13.2)	18 (16.8)	3 (8.3)	**<0.001**

BMI, body mass index; CABG, coronary artery bypass graft; CHD, coronary heart disease; CKD, chronic kidney disease; eGFR, estimated glomerular filtration rate; MI, myocardial infarction; NYHA, New York Heart Association; PCI, percutaneous coronary intervention; PAOD, peripheral artery occlusive disease; SD, standard deviation.

* determined by presence of a positive Sokolow Lyon index in the ECG.

a) Categories testet in J-T-test: non-diabetic patients (0) vs diabetic patients on dietetic (1), oral antidiabetic (2) or insulin (3) therapy.

b) Categories testet in J-T-test: patients receiving insulin therapy (3) vs patients on dietetic (1) or oral antidiabetic (2) therapy.

Patients’ age increased significantly with advanced renal impairment (p<0.001, Table 1). The percentage of women increased from 15.3% in the control group to 40.2% in CKD stage 4, and 36.1% in the CKD stage 5 group (p<0.001). Patients with proteinuria increased from 15.6% in CKD stage 2 to 69.2% in CKD stage 5 (p<0.001). The proportions of patients with traditional cardiovascular risk factors such as dyslipidemia (p = 0.08) and family history of CAD (p<0.001) decreased with progressive renal dysfunction, whereas diabetes mellitus and arterial hypertension were significantly more prevalent in patients with CKD stages 3 to 5 than in patients with mild renal impairment (both p<0.001).

Valvular heart disease was two to five times more often in patients with CKD stages 3, 4 and 5 than in the control group and in CKD stage 1 group (p<0.001, Table 1). There was no significant difference concerning mitral valve insufficiency, mitral valve stenosis, or aortic valve insufficiency between the CKD groups (data not shown) whereas significantly more patients with CKD 3–5 suffered from aortic valve stenosis compared to controls and patients with mild renal impairment (p = 0.003).

The percentage of patients with a history of previous stroke (p<0.001), previous MI (p = 0.02), previous coronary artery bypass grafting (CABG, p<0.001), previous PCI (p = 0.006), previous valve replacement (p<0.001), and implanted pacemaker (p<0.001) was significantly diverse between the different patient groups, with a trend to higher incidences among patients with progressive renal failure (Table 1).

Table 2 shows the basic laboratory parameters and reveals highly significant differences between the CKD subgroups (p<0.001). Spearman rank correlations are given in S1 Table demonstrating that the albumin/creatinine ratio as well as the protein/creatinine ratio were significantly positively associated with left ventricular ejection fraction (LVEF, p<0.001 and p = 0.003, respectively). The albumin/creatinine ratio was also significantly positively related to LVEF (when categorized as >40% and ≤40%, p = 0.001, S1 Table).

**Table 2:** Laboratory parameters.

	Overall population	Controls (eGFR ≥90 ml/min/1.73 m²), no proteinuria	CKD stage 1 (eGFR ≥90 ml/min/1.73 m²), proteinuria	CKD stage 2 (eGFR 60–89 ml/min/1.73 m²)	CKD stage 3 (eGFR 30–59 ml/min/1.73 m²)	CKD stage 4 (eGFR 15–29 ml/min/1.73 m²)	CKD stage 5 (eGFR <15 ml/min/1.73 m²) or dialysis	p
Patients, n (% of all)	3,352 (100.0)	629 (18.8)	127 (3.8)	1,599 (47.7)	854 (25.5)	107 (3.2)	36 (1.1)	
Creatinine, mean (95%CI), mg/dl	1.0 (1.0–1.0)	0.8 (0.8–0.8)	0.8 (0.8–0.8)	0.9 (0.9–0.9)	1.2 (1.2–1.2)	1.9 (1.8–2.0)	4.7 (3.9–5.6)	**<0.001**
eGFR	71.5 (70.8–72.3)	99.0 (98.5–99.5)	99.3 (98.1–100.5)	75.5 (75.1–75.9)	48.0 (47.4–48.5)	25.1 (24.3–25.9)	12.1 (9.8–14.3)	**<0.001**
Urea, mean (95%CI), mg/dl	21.7 (21.5–22.0)	17.8 (17.4–18.2)	19.4 (18.5–20.3)	20.1 (19.8–20.3)	26.5 (25.9–27.1)	41.3 (38.8–44.1)	49.9 (44.8–55.5)	**<0.001**
Albumin/creatinine, mean (95%CI), mg/g	33.8 (31.5–36.3)	18.5 (15.8–21.7)	25.0 (19.2–32.6)	26.4 (24.1–29.0)	50.5 (44.5–57.4)	121.9 (81.8–181.7)	277.6 (139.5–552.4)	**<0.001**
Protein/creatinine, mean (95%CI), mg/g	130.4 (126.7–134.2)	106.3 (100.7–112.1)	124.9 (108.4–144.0)	115.5 (111.3–119.9)	162.8 (153.8–172.4)	305.8 (241.4–387.5)	840.2 (497.9–1,417.9)	**<0.001**
Cholesterol, mean (95%CI), mg/dl	173.6 (172.1–175.1)	179.4 (176.2–182.7)	190.2 (181.7–199.1)	174.2 (172.1–176.4)	167.4 (164.4–170.4)	165.4 (156.0–175.4)	166.9 (154.2–180.7)	**<0.001**

eGFR indicates estimated glomerular filtration rate.

### Interventional parameters

**Fig 1:**
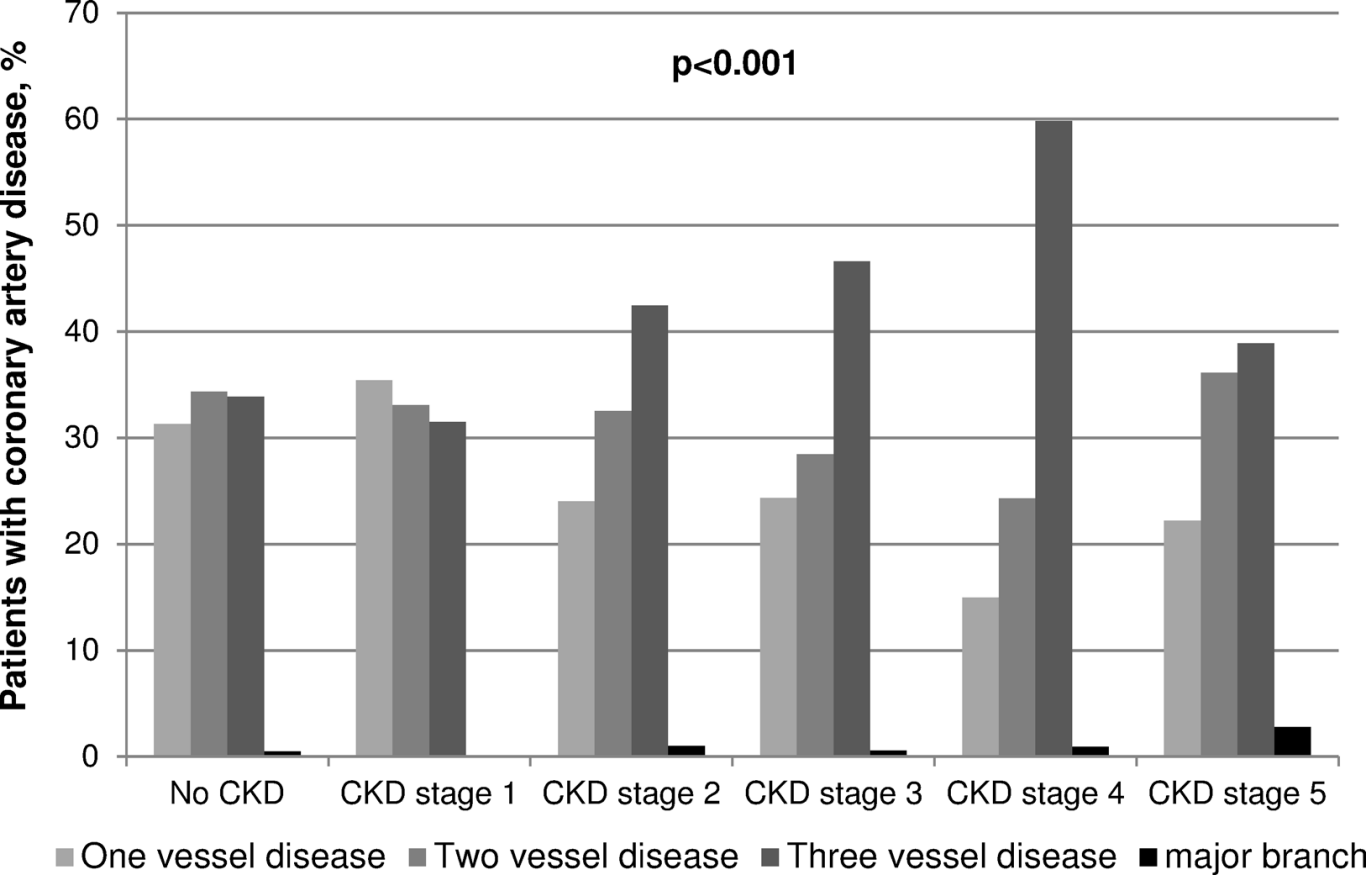
Proportions of patients with different degrees of CAD according to their renal function. P-values form Jonckheere-Terpstra tests.

In 16.4% to 42.5% of all coronary angiographies these were performed as emergency interventions due to an acute coronary syndrome (Table 3). In patients with CKD stage 1 the rate of emergency interventions was almost twice as high (42.5%; p<0.001, Table 3) compared to the average rate (21.3%). Additionally, patients with decreasing renal function suffered more often from coronary three vessel artery disease (p<0.001; Fig 1).

**Table 3:** Cardiological and angiographical data.

	Overall population	Controls (eGFR ≥90 ml/min/1.73 m²), no proteinuria	CKD stage 1 (eGFR ≥90 ml/min/1.73 m²), proteinuria	CKD stage 2 (eGFR 60–89 ml/min/1.73 m²)	CKD stage 3 (eGFR 30–59 ml/min/1.73 m²)	CKD stage 4 (eGFR 15–29 ml/min/1.73 m²)	CKD stage 5 (eGFR <15 ml/min/1.73 m²) or dialysis	p
Patients, n (% of all)	3,352 (100.0)	629 (18.8)	127 (3.8)	1,599 (47.7)	854 (25.5)	107 (3.2)	36 (1.1)	
Emergency angiography, n (%)	714 (21.3)	169 (26.9)	54 (42.5)	322 (20.1)	140 (16.4)	22 (20.6)	7 (19.4)	**<0.001**
Heart failure—none, n (%)	1,739 (55.9)	382 (67.0)	78 (67.8)	884 (59.7)	353 (43.6)	25 (24.8)	17 (47.2)	**<0.001**
NYHA I, n (%)	205 (6.6)	44 (7.7)	4 (3.5)	99 (6.7)	48 (5.9)	8 (7.9)	2 (5.6)	
NYHA II, n (%)	657 (21.1)	104 (18.2)	23 (20.0)	304 (20.5)	196 (24.2)	22 (21.8)	8 (22.2)	
NYHA III, n (%)	460 (14.8)	37 (6.5)	8 (7.0)	180 (12.2)	188 (23.2)	39 (38.6)	8 (22.2)	
NYHA IV, n (%)	52 (1.7)	3 (0.5)	2 (1.7)	14 (0.9)	25 (3.1)	7 (6.9)	1 (2.8)	
Coronary artery disease, n (%)	3,352							**<0.001**
one vessel disease	858 (25.6)	197 (31.3)	45 (35.4)	384 (24.0)	208 (24.4)	16 (15.0)	8 (22.2)	
two vessel disease	1,060 (31.6)	216 (34.3)	42 (33.1)	520 (32.5)	243 (28.5)	26 (24.3)	13 (36.1)	
three vessel disease	1,408 (42.0)	213 (33.9)	40 (31.5)	679 (42.5)	398 (46.6)	64 (59.8)	14 (38.9)	
main stem	26 (0.8)	3 (0.5)	0 (0.0)	16 (1.0)	5 (0.6)	1 (0.9)	1 (2.8)	
LVEF, n (%)	3,274							**<0.001**
Normal (>50%)	1,712 (52.3)	362 (58.7)	71 (55.9)	851 (54.7)	378 (45.4)	40 (37.7)	10 (27.8)	
Slightly reduced (41–50%)	621 (19.0)	118 (19.1)	15 (11.8)	298 (19.2)	167 (20.0)	15 (14.2)	8 (22.2)	
Moderately reduced (31–40%)	169 (5.2)	23 (3.7)	6 (4.7)	73 (4.7)	58 (7.0)	5 (4.7)	4 (11.1)	
Severely reduced (≤30%)	225 (6.9)	17 (2.8)	6 (4.7)	94 (6.0)	89 (10.7)	17 (16.0)	2 (5.6)	
PCI performed, n (%)	2,281 (68.0)	486 (77.3)	108 (85.0)	1,093 (68.4)	514 (60.2)	56 (52.3)	24 (66.7)	**<0.001**
Intervened arteries (LAD, LCX, RCA)	1,984 (59.2)	434 (69.0)	99 (78.0)	950 (59.4)	434 (50.8)	48 (44.9)	19 (52.8)	0.18
One, n (%)	1,788 (90.1)	397 (91.5)	91 (91.9)	853 (89.8)	389 (89.6)	41 (85.4)	17 (89.5)	
Two, n (%)	177 (8.9)	34 (7.8)	8 (98.1)	88 (9.3)	39 (9.0)	6 (12.5)	2 (10.5)	
Three, n (%)	19 (1.0)	3 (0.7)	0 (0.0)	9 (0.9)	6 (1.4)	1 (2.1)	0 (0.0)	
Performed stenting, n (%)	1,984	434 (92.7)	99 (95.2)	950 (91.3)	434 (90.4)	48 (88.9)	19 (90.5)	0.09
CABG recommended, n (%)	344 (10.3)	50 (7.9)	10 (7.9)	174 (10.9)	91 (10.7)	15 (14.0)	4 (11.1)	**0.04**

CABG, coronary artery bypass graft; CAD, coronary artery disease; CKD, chronic kidney disease; eGFR, estimated glomerular filtration rate; LAD, left artery descending; LVEF, left ventricular ejection fraction; LCX, left circumflex artery; PCI, percutaneous coronary intervention; RCA, right coronary artery; SD, standard deviation.

About two thirds of the patients with CKD stage 4 suffered from NYHA class II, III or IV (Table 3). Comparing patients with eGFR ≥60 ml/min/1.73 m^2^ and with patients with eGFR <60 ml/min/1.73 m^2^, the number of patients with decreased renal function (CKD stage 3–5) and NYHA class II, III, and IV was 1.2-fold, 2.4-fold and 4.1-fold that of patients with mildly reduced (CKD 1–2) or normal renal function (controls; Table 3).

CAD patients without (= controls) or mild renal impairment (= CKD stage 1) underwent a PCI more often than patients with moderate to severe renal impairment (p<0.001). In contrast, recommendations for CABG almost doubled in CKD stage 4 compared to the control group (p = 0.04; Table 3).

### Medication at enrollment and at discharge

**Fig 2:**
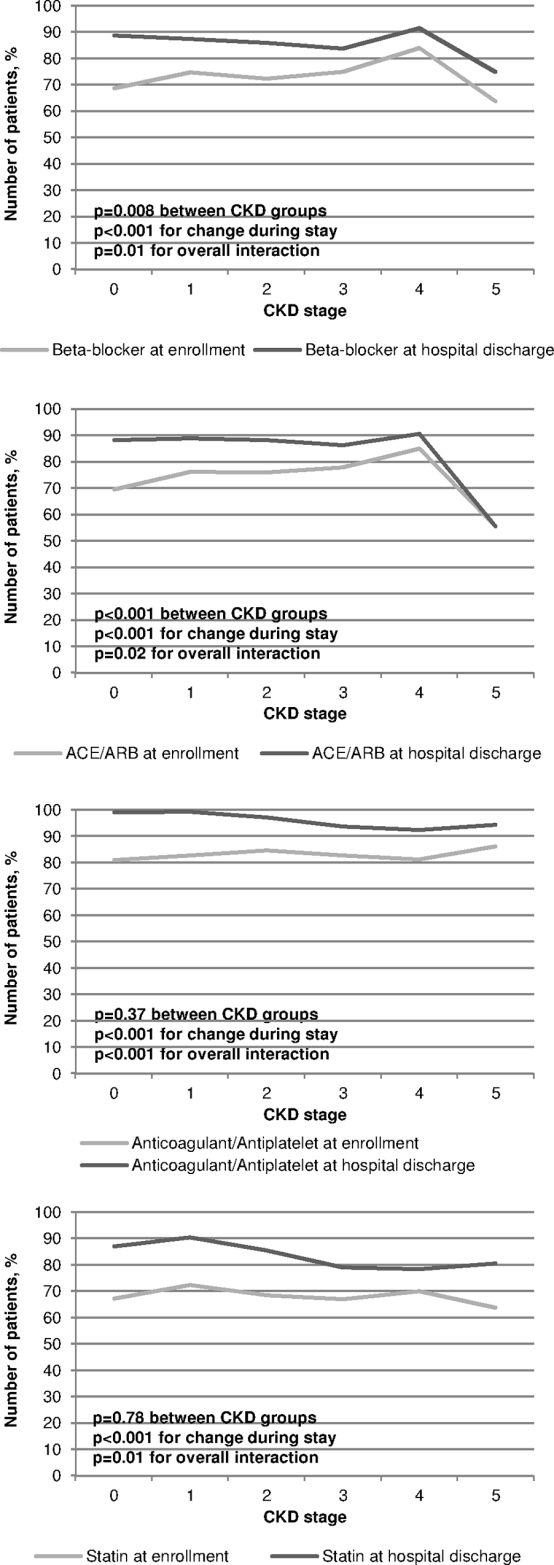
Proportion of patients (complete cohort) with respective medication prescription by CKD stage and visit (enrollment at hospital versus hospital discharge). Effects of CKD stage, visit and CKD stage x visit-interactions were tested via logistic regression.

There were marked changes regarding the medication between the time of enrollment (S2 Table) and discharge (S3 Table) depending on patients’ CKD stage. Fig 2 displays these differences between enrollment and discharge, as well as between the distinct CKD stages with regard to four standard drug classes in CAD (beta-blocker, angiotensin converting enzyme (ACE) inhibitors and/or angiotensin II type 1 receptor blockers (ARBs), anticoagulants, statins). Results of the corresponding logistic regression analyses are displayed in S4 Table. In summary, with regard to all four drug classes a significant increase in prescriptions between enrollment and discharge could be observed, as well as lower prescription rates of beta-blockers and ACE inhibitors/ARBs with increasing CKD stages. However, regarding all patients (Fig 2), significant interactions between CKD groups and visits were observed indicating that the probability for prescribing a distinct medication at a particular visit is associated with CKD stage.

**Fig 3:**
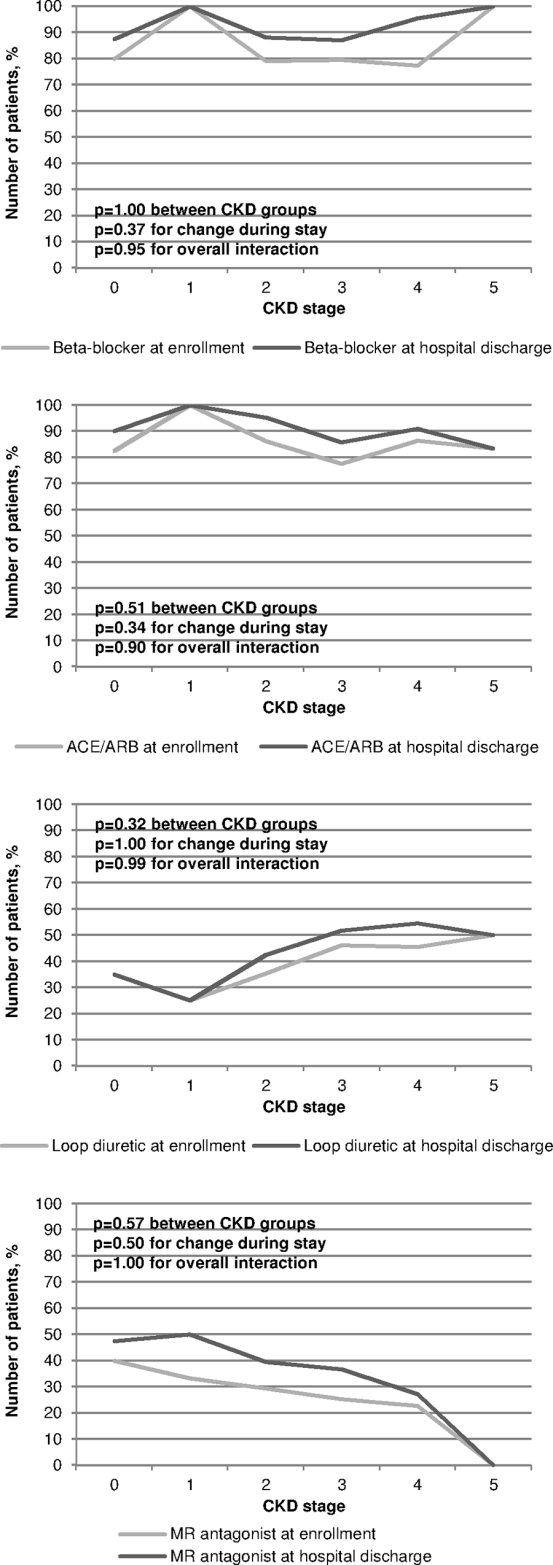
Proportion of patients (subgroup with moderately to severely reduced LVEF, LVEF≤40%) with respective medication prescription by CKD stage and visit (enrollment at hospital versus hospital discharge). Effects of CKD stage, visit and CKD stage x visit-interactions were tested via logistic regression.

In contrast, in the subgroup of patients with moderately to severely reduced LVEF the prescription frequency of beta-blockers and ACE inhibitors/ARBs was higher in patients with endstage renal disease as compared to the total cohort (Fig 3).

### In-hospital outcome

Five patients (0.1%) required dialysis after coronary intervention, 4 (11.1%) of them had been initially classified as patients in CKD stage 5. During or after intervention 7 (0.2%) patients suffered from new MI, and 5 patients (0.1%) suffered from stroke (Table 4). A recurrent PCI was necessary in 124 patients (3.7%); none of them was in the CKD stage 5 group. After coronary angiography, 98 patients (2.9%) underwent CABG, none of them in the CKD stage 5 group.

**Table 4:** In-hospital treatment and outcome.

	Overall population	Controls (eGFR ≥90 ml/min/1.73 m²), no proteinuria	CKD stage 1 (eGFR ≥90 ml/min/1.73 m²), proteinuria	CKD stage 2 (eGFR 60–89 ml/min/1.73 m²)	CKD stage 3 (eGFR 30–59 ml/min/1.73 m²)	CKD stage 4 (eGFR 15–29 ml/min/1.73 m²)	CKD stage 5 (eGFR <15 ml/min/1.73 m²) or dialysis	p^a)^
Patients, n (% of all)	3,352 (100.0)	629 (18.8)	127 (3.8)	1,599 (47.7)	854 (25.5)	107 (3.2)	36 (1.1)	
CABG, n (%)	98 (2.9)	14 (2.2)	5 (3.9)	47 (2.9)	26 (3.0)	6 (5.7)	0 (0.0)	0.32
MI after angiography, n (%)	7 (0.2)	4 (0.6)	0 (0.0)	2 (0.1)	0 (0.0)	0 (0.0)	1 (2.8)	0.09
Re-PCI, n (%)	124 (3.7)	27 (4.3)	3 (2.4)	66 (4.1)	26 (3.0)	2 (1.9)	0 (0.0)	0.11
Stroke, n (%)	5 (0.1)	2 (0.3)	0 (0.0)	3 (0.2)	0 (0.0)	0 (0.0)	0 (0.0)	0.13
Discharge status								**0.03**
Discharged to home, n (%)	3,037 (90.7)	581 (92.5)	113 (89.0)	1,448 (90.6)	777 (91.0)	86 (80.4)	32 (88.9)	0.06
In-hospital death, n (%)	8 (0.2)	0 (0.0)	0 (0.0)	3 (0.2)	3 (0.4)	1 (0.9)	1 (2.8)	**0.02**
Discharged to another hospital, n (%)	263 (7.9)	42 (6.7)	7 (5.5)	129 (8.1)	66 (7.7)	17 (15.9)	2 (5.6)	0.09
Discharge to rehabilitation measures, n (%)	42 (1.3)	5 (0.8)	7 (5.5)	18 (1.1)	8 (0.9)	3 (2.8)	1 (2.8)	0.92

CABG, coronary artery bypass graft; CKD, chronic kidney disease; eGFR, estimated glomerular filtration rate; MI, myocardial infarction; PCI, percutaneous coronary intervention.

^a)^ p-values for variables CABG, MI after angiography, Re-PCI and stroke are from J-T-tests; p-values for discharge status result from a Kruskal-Wallis-test followed by category-wise Mann-Whitney-U-tests.

**Fig 4:**
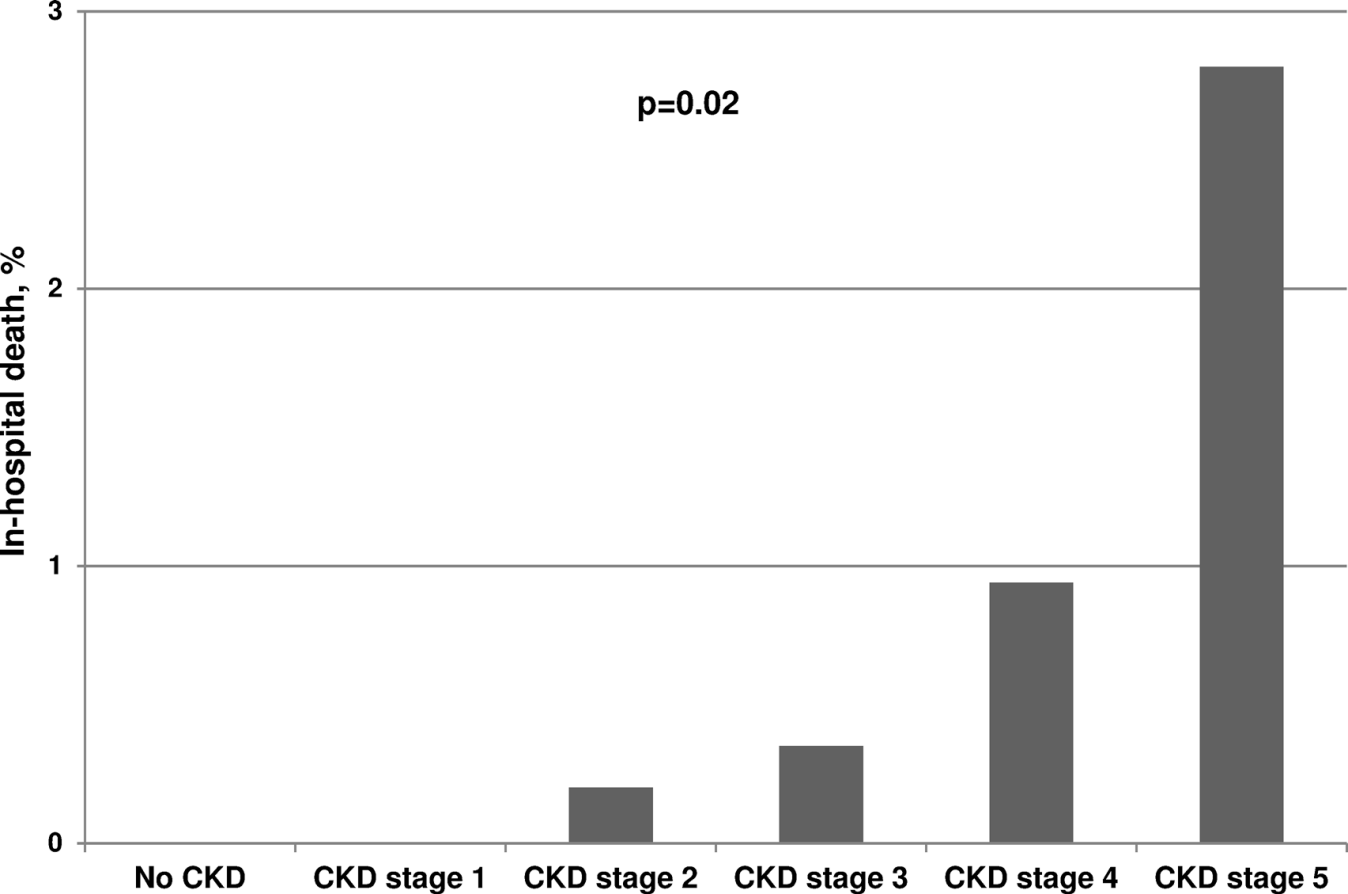
Proportion of patients who died in-hospital according to their renal function. P-value from Mann-Whitney-U-test testing category “In-hospital death” vs all other categories of variable “discharge status”.

A total of 3,037 (90.6%) patients were discharged alive, 263 (7.9%) were transferred to another hospital, and 42 (1.3%) were discharged to medical rehabilitation measures (Table 4). Eight (0.2%) patients died during hospital stay with a continuously increasing mortality in patients with progressive renal impairment (Table 4, Fig 4).

## Discussion

The CAD-REF registry is a German-wide prospective observational multicenter registry. It aims at evaluating the characteristics and outcome of patients with angiographically documented CAD, with and without concomitant CKD to identify new risk factors and biomarkers for patients’ prognosis. Another important issue is the evaluation of current invasive treatment patterns and prescription rates of guideline-recommended standard medication.

Until now, only few prospective observational studies exist that evaluate treatment, outcome and factors contributing to the progression of CKD [18–20,25]. Of these, only CAD-REF and APPROACH [25] included a control group with preserved renal function. Moreover, only the CAD-REF registry assessed the coronary status determined by coronary angiography in detail (classification based on CARDS [24]).

In the CAD-REF registry, we found that with advanced renal impairment known cardiovascular risk factors such as hypertension and diabetes mellitus were significantly more frequent. Many other comorbidities, such as previous MI and CABG, valve replacement and advanced stages of congestive heart failure were also increased with advanced renal failure. Our findings are in line with other studies demonstrating the prominent cardiovascular morbidity and mortality of CKD patients [4–6,8,15,26, 27]. Moreover, based on our detailed angiographic evaluation we were able to show that the clinical severity of CAD strongly depended on residual eGFR and thus patients’ CKD stages, with a higher number of diseased coronary vessels (Fig 1) and a lower LVEF with declining eGFRs (Table 3). Two other studies also described an increase in severity of CAD with impaired renal function, although both studies were based on a case control design of non-CKD and CKD patients with eGFR above or below 60 ml/min/1.73 m^2^ [28,29].

Furthermore, we found highly significant correlations between angiographic parameters and the urinary albumin/creatinine and protein/creatinine ratio (S1 Table). Our results point to the fact that proteinuria in combination with other risk scores (FRAMINGHAM; PROCAM) may enhance the sensitivity and specificity for predicting the outcome of patients with and without known CAD. The planned follow-up analyses in CAD-REF will show whether and to what extent the combination of the clinical, angiographic and laboratory parameters will help to identify different risk groups and identify patients at high cardiovascular risk.

### Differences in in-hospital treatment

At the time of enrollment a relatively low number of CAD-REF patients were treated with cardioprotective drugs such as ACE inhibitors or ARBs, beta-blockers, statins and platelet inhibitors as also found in other reports [14,15,27,30,31,]. From enrollment to discharge, these treatments increased markedly in patients with CKD stage 1 to 3, but no comparable increase was observed in stage 4 and 5; the latter received especially ACE inhibitors and ARBs but also beta-blockers and statins significantly less frequently (Fig 2). This finding is remarkable since ACE inhibitors and ARBs not only belong to the standard therapy for hypertension and CAD, but are also known to slow down kidney disease progression and to improve proteinuria in diabetic nephropathy [16,32]. Therefore, current guidelines give clear recommendations in favor of these drugs even in higher CKD stages [16,33].

Medical undertreatment, known as “therapeutic nihilism” in patients with CAD and CKD has been reported previously in other cardiological settings [14,15,27]. This observation might partly be explained by cardiologists’ concerns to induce further kidney deterioration by these drugs. In contrast, in cohorts mainly treated by nephrologists [30,34,35] the use of ACE inhibitors, ARBs and loop diuretics was markedly higher than in our registry, and moreover further increased with higher CKD stages. Vice versa, the use of beta-blockers was 2 to 3 times higher in our series compared to the nephrological studies [30,34,35] demonstrating that cardiologists still have a favor for these substances.

Interestingly, in CAD-REF patients with a LVEF ≤40% the prescription of beta-blockers, ACE inhibitors and ARBs, and loop diuretics was higher in all CKD stages including stage 4 and 5 (Fig 3) compared to the whole cohort, which may indicate that cardiologists ranked the treatment of heart failure above potential harms on kidney function.

Therefore, more effort is needed to promote the current KDIGO guidelines to other disciplines beside nephrology to underline that ACE inhibitors and ARBs are also nephroprotective in all stages of CKD.

In patients with severe heart failure (NYHA class III-IV), also aldosterone receptor antagonists (MR antagonists) are recommended. The ACCF/AHA 2013 guidelines [36] also recommend the use of MR antagonists in patients with NYHA class II and LVEF ≤35% who have a history of prior cardiovascular hospitalization or elevated plasma natriuretic peptide levels, and postmyocardial infarction patients with LVEF ≤40% who develop heart failure symptoms or have a history of diabetes mellitus (ACCF/AHA). However, in our study only 7.0% of the overall study population was treated with a MR antagonist (spironolactone or eplerenone) while 9.3% of the CKD stage 4 patients and 2.8% of the CKD stage 5 patients received this medication. This is of relevance as dosing of spironolactone has to be adapted accordingly in patients with heart failure and concomitant renal impairment and is explicitly not recommended in patients with an eGFR <30 ml/minute/1.73 m^2^ (ACCF/AHA) [36].

### Limitations and conclusion

Our data derived from an observational registry and not from a randomized study and allow therefore no conclusions about causal interactions. However, our data reflect the current treatment patterns in an industrialized nation in which all inhabitants are insured by law and all health expenses are reimbursed which, therefore, cannot represent a reason for low prescriptions of drugs.

The number of patients in CKD stage 4 and 5 was rather small with only 143 patients. This weakens statements for this subgroup. Due to the “real world design” of the study with consecutive recruitment without patients’ preselection the composition of the study cohort illustrates a representative sample of the German population with CAD and concomitant CKD.

In summary, CAD patients with decreasing renal function not only suffer from more comorbidities but also receive standard treatment still less often in contrast to current guideline recommendations. Since CKD advances to a global health problem, and cardiologists and nephrologists have different treatment concepts for the same patients at risk, more consequent and in part more common treatment strategies and a broader knowledge of them appear to be recommended to meet the challenges of this disease burden.

## Supporting Information

S1 Fig Distribution of patients in the CAD-REF registry according to CKD stage. (TIF)

S1 File Definition of cardiovascular risk factors. (PDF)

S1 Table Spearman rank correlational analysis of laboratory parameters and coronary artery disease, multivessel disease, LVEF and LVEF≤40%. (PDF)

S2 Table Medication at time of enrollment before coronary angiography. (PDF)

S3 Table Medication at hospital discharge after coronary angiography. (PDF)

S4 Table Logistic regression for effects of CKD group, visits and interaction with regard to medication in the complete CAD-REF cohort. (PDF)
