# Supplementary material for: Baseline Characteristics and Prescription Patterns of Standard Drugs in Patients with Angiographically Determined Coronary Artery Disease and Renal Failure (CAD-REF Registry)
Source: PLoS One. 2016 Feb 9;11(2):e0148057. doi: 10.1371/journal.pone.0148057 (PMC4747471; doi:10.1371/journal.pone.0148057)
Supplement: S1 File — (PDF) [file pone.0148057.s002.pdf]

**S1 File. Definition of cardiovascular risk factors.**

A detailed trial design is reported elsewhere [1]. Hyperlipidaemia was defined as a total plasma cholesterol >5.2 mmol/l (>200 mg/dl), plasma LDL-cholesterol >3.87 mmol/l (>150 mg/dl), or plasma lipoprotein (a) >250 mg/l. Diabetes was assumed if patients required a specific diet, took oral antidiabetic medication, or received insulin. Regular physical activity was defined by less than 3 times, 3 to 5 times, or more than 5 times weekly physical activity for at least 30 minutes; low physical activity was defined as occasional or irregular physical activity. Alcohol consumption was assumed if patients consumed occasionally, regularly or excessively alcohol. Peripheral artery occlusive disease was divided into the 4 stages according to the Fontaine classification [2]. Family history of chronic artery disease was assumed if parents, siblings, or children suffered from previous myocardial infarction, stroke, coronary artery bypass graft, or stent implantation. Left ventricular hypertrophy was defined by a positive Sokolow-Lyon index in the electrocardiogram (ECG) [3].

**References**


1. Brand E, Pavenstädt H, Schmieder RE, Engelbertz C, Fobker M, Pinnschmidt HO, et al. The Coronary Artery Disease and Renal Failure (CAD-REF) registry: trial design, methods, and aims. Am Heart J. 2013;166:449-456.
2. Fontaine R, Kim M, Kieny R. Surgical treatment of peripheral circulation disorders. Helv Chir Acta. 1954;21:499-533.
3. Sokolow M, Lyon TP. The ventricular complex in left ventricular hypertrophy as obtained by unipolar precordial and limb leads. Am Heart J. 1949;37:161-186.
