# Supplementary material for: Baseline Characteristics and Prescription Patterns of Standard Drugs in Patients with Angiographically Determined Coronary Artery Disease and Renal Failure (CAD-REF Registry)
Source: PLoS One. 2016 Feb 9;11(2):e0148057. doi: 10.1371/journal.pone.0148057 (PMC4747471; doi:10.1371/journal.pone.0148057)
Supplement: S1 Table — (PDF) [file pone.0148057.s003.pdf]

S1 Table: Spearman rank correlational analysis of laboratory parameters and coronary artery disease, multivessel disease, LVEF and LVEF≤40%.

Correlation of albumin/creatinine ratio with	Correlation coefficient ρ	p-value
Coronary artery disease	0.038	0.1
Multivessel disease	0.025	0.3
LVEF	0.101	**<0.001**
LVEF ≤40%	0.089	**0.001**

Correlation of protein/creatinine ratio with	Correlation coefficient ρ	p-value
Coronary artery disease	0.033	0.07
Multivessel disease	0.016	0.4
LVEF	0.059	**0.003**
LVEF ≤40%	0.036	0.07

LVEF indicates left ventricular ejection fraction.

Coronary artery disease composes of one-, two-, three-vessel disease and main stem stenosis; multivessel disease composes of two- and three-vessel disease and main stem; LVEF is composes of the categories normal (>50%), slightly reduced (41-50%), moderately reduced (31-40%) and severely reduced (≤30%) LVEF, LVEF ≤40% is composed of the categories normal or slightly reduced (>40%) and moderately to severely reduced (≤40%) LVEF.
