# Supplementary material for: Baseline Characteristics and Prescription Patterns of Standard Drugs in Patients with Angiographically Determined Coronary Artery Disease and Renal Failure (CAD-REF Registry)
Source: PLoS One. 2016 Feb 9;11(2):e0148057. doi: 10.1371/journal.pone.0148057 (PMC4747471; doi:10.1371/journal.pone.0148057)
Supplement: S2 Table — (PDF) [file pone.0148057.s004.pdf]

S2 Table: Medication at time of enrollment before coronary angiography.

	**Overall population**	**No CKD** (eGFR ≥90 ml/min/1.73 m²), no proteinuria	**CKD stage 1** (eGFR ≥90 ml/min/1.73 m²), proteinuria	**CKD stage 2** (eGFR 60-89 ml/min/1.73 m²)	**CKD stage 3** (eGFR 30-59 ml/min/1.73 m²)	**CKD stage 4** (eGFR 15-29 ml/min/1.73 m²)	**CKD stage 5** (eGFR <15 ml/min/1.73 m²) or on haemodialysis
Patients, n (% of all)	3,352 (100.0)	629 (18.8)	127 (3.8)	1,599 (47.7)	854 (25.5)	107 (3.2)	36 (1.1)
Antihypertensive drug, all, n (%)	3,013 (89.9)	520 (82.7)	114 (89.8)	1,441 (90.1)	804 (94.1)	105 (98.1)	29 (80.6)
ACE inhibitor and/or AT1 receptor blocker, n (%)	2,528 (75.4)	438 (69.6)	97 (76.4)	1,215 (76.0)	667 (78.1)	91 (85.0)	20 (55.6)
ACE inhibitor, n (%)	1,902 (56.7)	373 (59.3)	79 (62.2)	912 (57.0)	467 (54.7)	58 (54.2)	13 (36.1)
AT1 receptor blocker, n (%)	685 (20.4)	74 (11.8)	21 (16.5)	328 (20.5)	217 (25.4)	36 (33.6)	9 (25.0)
Beta-blocker, n (%)	2,439 (72.8)	433 (68.8)	95 (74.8)	1,157 (72.4)	641 (75.1)	90 (84.1)	23 (63.9)
Calcium channel blocker, n (%)	258 (7.7)	33 (5.2)	7 (5.5)	134 (8.4)	75 (8.8)	7 (6.5)	2 (5.6)
Loop diuretic, n (%)	625 (18.6)	44 (7.0)	12 (9.4)	220 (13.8)	275 (32.2)	57 (53.3)	17 (47.2)
Diuretic, other (thiazides, potassium-sparing), n (%)	1,178 (35.1)	164 (26.1)	46 (36.2)	549 (34.3)	372 (43.6)	36 (33.6)	11 (30.6)
Centrally acting antihypertensive drug, n (%)	90 (2.7)	3 (0.5)	2 (1.6)	34 (2.1)	38 (4.4)	10 (9.3)	3 (8.3)
Alpha-blocker, n (%)	61 (1.8)	6 (1.0)	1 (0.8)	25 (1.6)	21 (2.5)	5 (4.7)	3 (8.3)

Anticoagulant + Antiplatelet, n (%)	2,794 (83.1)	510 (81.1)	105 (82.7)	1,355 (84.7)	706 (82.7)	87 (81.3)	31 (86.1)
Platelet aggregation inhibitor ASA, n (%)	2,569 (76.6)	484 (76.9)	103 (81.1)	1,251 (78.2)	626 (73.3)	78 (72.9)	27 (75.0)
Anticoagulant, vitamin K-antagonist, n (%)	182 (5.4)	11 (1.7)	0 (0.0)	76 (4.8)	84 (9.8)	8 (7.5)	3 (8.3)
Statin (HMG-CoA reductase inhibitor), n (%)	2,282 (68.1)	423 (67.2)	92 (72.4)	1,097 (68.6)	572 (67.0)	75 (70.1)	23 (63.9)
Fibrate, n (%)	19 (0.6)	1 (0.2)	0 (0.0)	8 (0.5)	8 (0.9)	2 (1.9)	0 (0.0)
Antidiabetic drug, all, n (%)	719 (21.4)	87 (13.8)	24 (18.9)	320 (20.0)	238 (27.9)	38 (35.5)	12 (33.3)
Antidiabetic drug insulin, n (%)	340 (10.1)	42 (6.7)	10 (7.9)	128 (8.0)	122 (14.3)	26 (24.3)	12 (33.3)
MR antagonist, n (%)	235 (7.0)	36 (5.7)	9 (7.1)	99 (6.2)	80 (9.4)	10 (9.3)	1 (2.8)

ACE: angiotensin converting enzyme; ASA: acetylsalicylic acid; AT1: angiotensin II; HMG-CoA: 3-hydroxy-3-methylglutaryl-coenzyme A; MR: mineralocorticoid receptor
