# Supplementary material for: Baseline Characteristics and Prescription Patterns of Standard Drugs in Patients with Angiographically Determined Coronary Artery Disease and Renal Failure (CAD-REF Registry)
Source: PLoS One. 2016 Feb 9;11(2):e0148057. doi: 10.1371/journal.pone.0148057 (PMC4747471; doi:10.1371/journal.pone.0148057)
Supplement: S3 Table — (PDF) [file pone.0148057.s005.pdf]

S3 Table: Medication at hospital discharge after coronary angiography.

	**Overall population**	**No CKD** (eGFR ≥90 ml/min/1.73 m²), no proteinuria	**CKD stage 1** (eGFR ≥90 ml/min/1.73 m²), proteinuria	**CKD stage 2** (eGFR 60-89 ml/min/1.73 m²)	**CKD stage 3** (eGFR 30-59 ml/min/1.73 m²)	**CKD stage 4** (eGFR 15-29 ml/min/1.73 m²)	**CKD stage 5** (eGFR <15 ml/min/1.73 m²) or on haemodialysis
Patients, n (% of all)	3,352 (100.0)	629 (18.8)	127 (3.8)	1,599 (47.7)	854 (25.5)	107 (3.2)	36 (1.1)
Antihypertensive drug, all, n (%)	3,287 (98.1)	618 (98.3)	126 (99.2)	1,567 (98.0)	839 (98.2)	106 (99.1)	31 (86.1)
ACE inhibitor and/or AT1 receptor blocker, n (%)	2,935 (87.6)	556 (88.4)	113 (89.0)	1,411 (88.2)	738 (86.4)	97 (90.7)	20 (55.6)
ACE inhibitor, n (%)	2,299 (68.6)	491 (78.1)	98 (77.2)	1,100 (68.8)	533 (62.4)	64 (59.8)	13 (36.1)
AT1 receptor blocker, n (%)	742 (22.1)	79 (12.6)	23 (18.1)	360 (22.5)	231 (27.0)	40 (37.4)	9 (25.0)
Beta-blocker, n (%)	2,884 (86.0)	558 (88.7)	111 (87.4)	1,374 (85.9)	716 (83.8)	98 (91.6)	27 (75.0)
Calcium channel blocker, n (%)	305 (9.1)	39 (6.2)	9 (7.1)	157 (9.8)	88 (10.3)	9 (8.4)	3 (8.3)
Loop diuretic, n (%)	723 (21.6)	55 (8.7)	13 (10.2)	266 (16.6)	306 (35.8)	65 (60.7)	18 (50.0)
Diuretic, other (thiazides, potassium-sparing), n (%)	1,358 (40.5)	192 (30.5)	57 (44.9)	639 (40.0)	421 (49.3)	38 (35.5)	11 (30.6)
Centrally acting antihypertensive drug, n (%)	100 (3.0)	4 (0.6)	3 (2.4)	37 (2.3)	42 (4.9)	11 (10.3)	3 (8.3)

Alpha-blocker, n (%)	68 (2.0)	9 (1.4)	1 (0.8)	25 (1.6)	24 (2.8)	6 (5.6)	3 (8.3)
Anticoagulant + Antiplatelet, n (%)	3,237 (96.6)	623 (99.0)	126 (99.2)	1,554 (97.2)	801 (93.8)	99 (92.5)	34 (94.4)
Platelet aggregation inhibitor ASA, n (%)	3,062 (91.3)	609 (96.8)	124 (97.6)	1,477 (92.4)	734 (85.9)	90 (84.1)	28 (77.8)
Anticoagulant, vitamin K-antagonist, n (%)	281 (8.4)	21 (3.3)	3 (2.4)	122 (7.6)	117 (13.7)	14 (13.1)	4 (11.1)
Statin (HMG-CoA reductase inhibitor), n (%)	2,856 (85.2)	555 (88.2)	118 (92.9)	1,383 (86.5)	687 (80.4)	84 (78.5)	29 (80.6)
Fibrate, n (%)	25 (0.7)	1 (0.2)	2 (1.6)	11 (0.7)	9 (1.1)	2 (1.9)	0 (0.0)
Antidiabetic drug, all, n (%)	806 (24.0)	103 (16.4)	29 (22.8)	353 (22.1)	267 (31.3)	41 (38.3)	13 (36.1)
Antidiabetic drug insulin, n (%)	382 (11.4)	46 (7.3)	13 (10.2)	141 (8.8)	140 (16.4)	29 (27.1)	13 (36.1)
MR antagonist, n (%)	322 (9.6)	49 (7.8)	16 (12.6)	139 (8.7)	106 (12.4)	11 (10.2)	1 (2.8)

ACE: angiotensin converting enzyme; ASA: acetylsalicylic acid; AT1: angiotensin II; HMG-CoA: 3-hydroxy-3-methylglutaryl-coenzyme A; MR: mineralocorticoid receptor
