# Supplementary material for: Baseline Characteristics and Prescription Patterns of Standard Drugs in Patients with Angiographically Determined Coronary Artery Disease and Renal Failure (CAD-REF Registry)
Source: PLoS One. 2016 Feb 9;11(2):e0148057. doi: 10.1371/journal.pone.0148057 (PMC4747471; doi:10.1371/journal.pone.0148057)
Supplement: S4 Table — (PDF) [file pone.0148057.s006.pdf]

S4 Table: Logistic regression for effects of CKD group, visits and interaction with regard to medication in the complete CAD-REF cohort.

	p-value CKD group	p-value visit	p-value interaction
Antihypertensive drug, all	**<0.001**	**<0.001**	**0.02**
ACE inhibitor and/or AT1 receptor blocker	**<0.001**	**<0.001**	**0.02**
ACE inhibitor	0.05	**<0.001**	**0.008**
AT1 receptor blocker	**<0.001**	0.7	1.00
Beta-blocker	**0.008**	**<0.001**	**0.01**
Calcium channel blocker	0.1	0.5	1.00
Loop diuretic	**<0.001**	0.3	0.9
Diuretic, other (thiazides, potassium-sparing)	**<0.001**	0.08	0.9
Centrally acting antihypertensive drug	**<0.001**	0.7	1.00
Alpha-blocker	**0.006**	0.4	0.9
Anticoagulant + Antiplatelet	0.4	**<0.001**	**<0.001**
Platelet aggregation inhibitor ASA	0.08	**<0.001**	**<0.001**
Anticoagulants, vitamin K-antagonist	**<0.001**	0.08	0.9
Statin (HMG-CoA reductase inhibitor)	0.8	**<0.001**	**0.01**
Fibrate	0.3	1.00	1.00
Antidiabetic drug, all	**<0.001**	0.2	1.00
Antidiabetic drug insulin	**<0.001**	0.7	1.00
MR antagonist	**0.03**	0.2	0.9

ACE: angiotensin converting enzyme; ASA: acetylsalicylic acid; AT1: angiotensin II; HMG-CoA: 3-hydroxy-3-methylglutaryl-coenzyme A; MR: mineralocorticoid receptor
